# 343. RURAL-COVID-19 Trial: Retrospective Analysis of COVID-19 Coinfections in Hospitalized Urban and Rural Adults

**DOI:** 10.1093/ofid/ofab466.544

**Published:** 2021-12-04

**Authors:** Caroline Hamilton, Heather Frazier, Jose A Vazquez

**Affiliations:** 1 Augusta University/Medical College of Georgia, Augusta, Georgia; 2 Augusta University Medical Center, Augusta, Georgia; 3 Medical College of Georgia at Augusta University, Augusta, Georgia

## Abstract

**Background:**

The impact of COVID-19 in rural communities has been well described. However, little is known regarding differences in coinfections among COVID-19 patients in rural vs. urban settings. Our primary objective is to evaluate community acquired coinfection (CACo) rates (< 72 hrs from admission) and healthcare-associated infection (HAI) rates ( > 72 hrs from admission) in these populations. Secondary objectives include use of empiric antibiotics, pathogen prevalence, and patient outcomes.

**Methods:**

Retrospective analysis of the first 255 adult patients admitted to a tertiary medical center with symptomatic COVID-19 and confirmed by PCR. Rural and urban categories were determined using patient address and county census data. Isolated pathogens were individually evaluated and considered coinfections if the patient met predetermined criteria.

Predetermined Coinfection Criteria

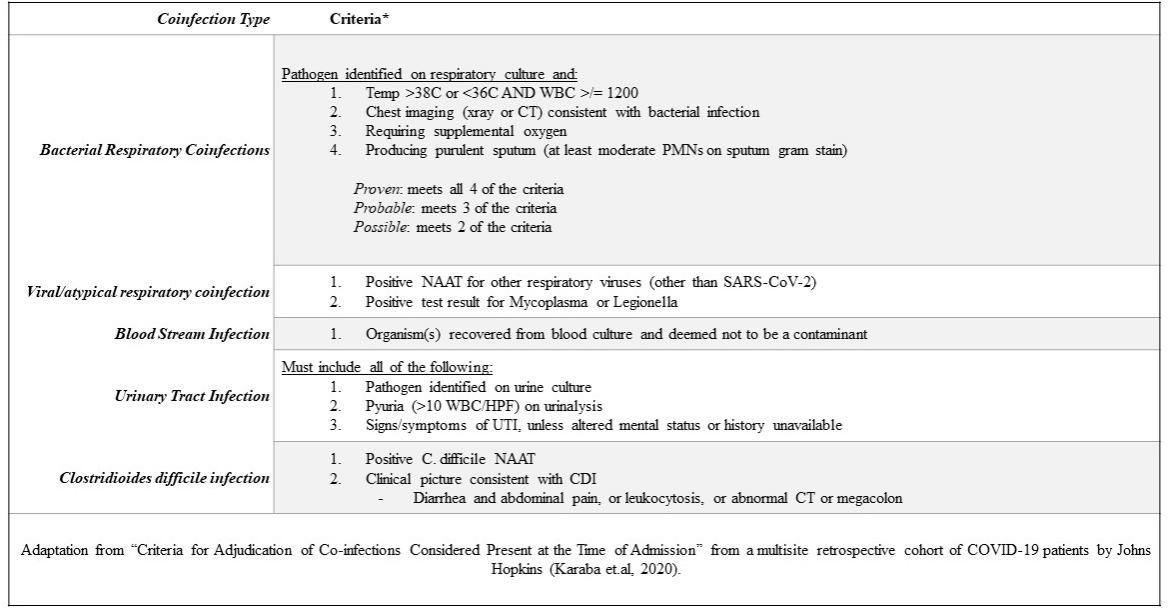

**Results:**

The rates of CACo for rural (n = 90) and urban (n = 165) residents were 11.1% and 13.3%, respectively. Non-respiratory coinfections, such as bloodstream and urinary tract infections, were more common in urban residents; however, empiric antibiotics were started in 75.1% of all subjects. *Methicillin susceptible staphylococcus aureus* and *Escherichia coli* were the most common pathogens isolated on admission in both populations. HAI rates were 13.3% in the rural residents vs 13.9% in the urban residents with *Methicillin resistant staphylococcus aureus* as the most common respiratory pathogen, although *Pseudomonas aeruginosa* was the most prevalent overall pathogen. There was no significant difference in hospital length of stay or 30-day all-cause mortality among both populations.

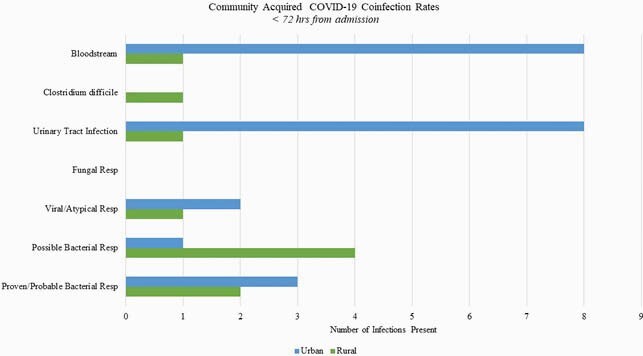

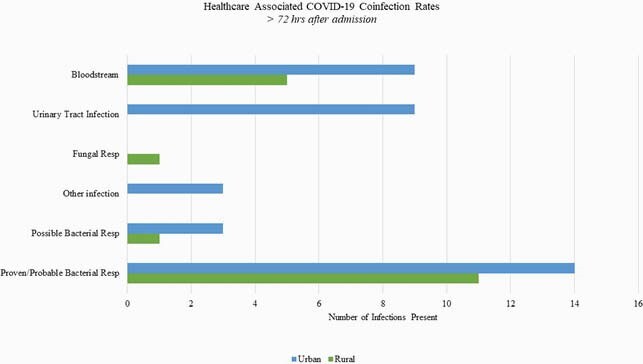

Patient Outcomes Among Rural and Urban Populations

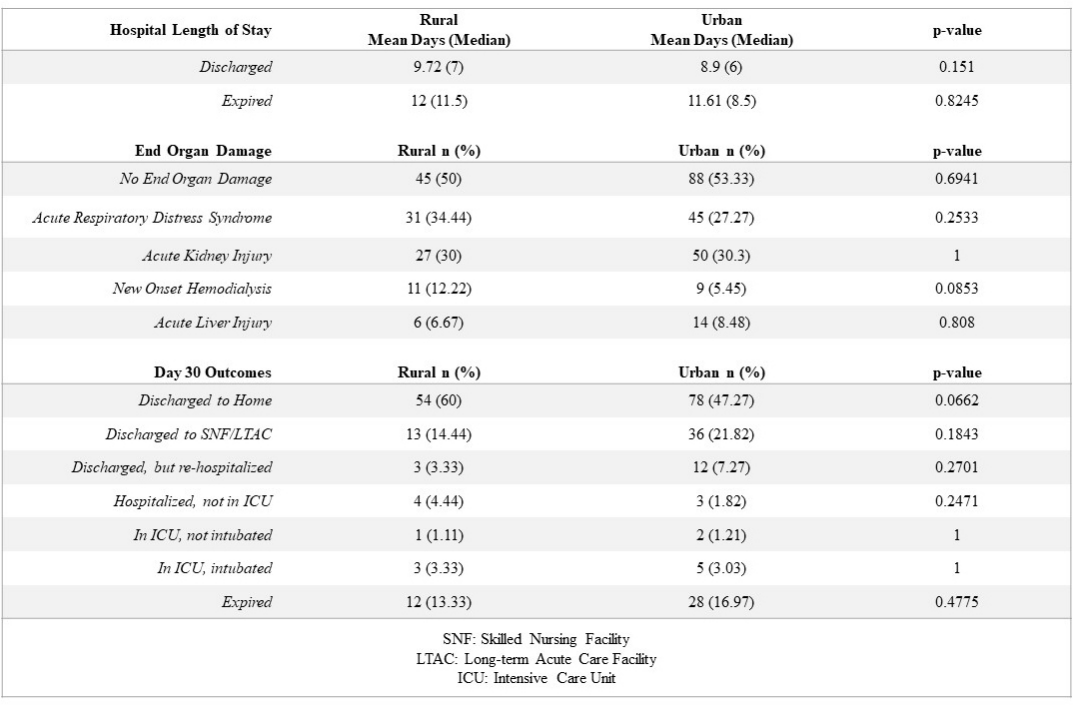

**Conclusion:**

There was no significant difference in the rate of CACo or HAI among rural or urban populations. Despite the high rate of antibiotic use to empirically cover community acquired respiratory infections at the start of the pandemic, only 1.9% of the subjects had a possible or proven respiratory coinfection on admission. Despite prior research showing worse outcomes for rural populations with COVID-19, our data demonstrates that coinfection rates and patient outcomes were similar among these populations when receiving medical care at an academic hospital.

**Disclosures:**

**All Authors**: No reported disclosures

